# Factors influencing adherence to positive airway pressure therapy in stroke patients with obstructive sleep apnea: a cross-sectional study

**DOI:** 10.3389/fneur.2025.1604935

**Published:** 2025-06-20

**Authors:** Wen-Yi Yu, Li-Wen Xu, Shu-Tong Sun, Yi-Xi Zheng, Tian-Yu Jing, Gang Xu, Tie-Yu Tang, Cheng Chu

**Affiliations:** ^1^Department of Neurology, The Affiliated Hospital of Yangzhou University, Yangzhou University, Yangzhou, China; ^2^School of Nursing and School of Public Health, Yangzhou University, Yangzhou, China

**Keywords:** cerebral stroke, obstructive sleep apnea, CPAP ventilation, treatment adherence, health belief models

## Abstract

**Background:**

Positive Airway Pressure (PAP) treatment is the recommended initial approach for moderately severe obstructive sleep apnea patients. Its efficacy is contingent upon patient compliance, yet compliance studies in combined stroke and obstructive sleep apnea (OSA) patients have demonstrated lower rates of compliance, and most of the influencing factors are unregulated. This study aimed to explore short-term respiratory therapy compliance status among stroke patients with obstructive sleep apnea and identify modifiable influencing factors to improve compliance and create personalized plans.

**Methods:**

This study was conducted among 254 stroke patients with OSA. Data were collected using standardized questionnaires, including the Pittsburgh Sleep Quality Index (PSQI), Epworth Sleepiness Scale (ESS), and Self-Efficacy Measure for Sleep Apnea (SEMSA). Polysomnography (PSG) was used to assess objective sleep parameters. Logistic regression analysis was performed to identify predictors of PAP adherence.

**Results:**

The overall compliance rate of stroke patients with OSA was 27.2%, and self-efficacy in patients with stroke combined with OSA (perceived risk (OR = 2.23, 95% CI = 1.74 ~ 2.83), expected effect of treatment (OR = 1.23, 95% CI = 1.23 ~ 1.4), self-assessment (OR = 1.17, 95% CI = 1.06 ~ 1.30), total score on the Health Beliefs Scale (OR = 1.20, 95% CI = 1.13 ~ 1.26)), objective sleep condition (total sleep duration (OR = 1.00, 95% CI = 1.00 ~ 1.01), sleep efficiency (OR = 1.00, 95% CI = 1.00 ~ 1.04)) (OR = 1.01, 95% CI = 1.00 ~ 1.02), N1 phase duration (OR = 1.01, 95% CI = 1.00 ~ 1.01)), OSA severity (AHI (OR = 1.04, 95% CI = 1.02 ~ 1.06), and longest hypoventilation time (s) (OR = 1.02, 95% CI = 1.00 ~ 1.03), and oxygen desaturation ≥3 index (ODI) (OR = 1.03, 95% CI = 1.01 ~ 1.05) were the risk factors affecting their PAP treatment.

**Conclusion:**

Patients with stroke combined with OSA have poorer compliance to PAP treatment (27.2%) compared with the general population, and this compliance is closely related to self-efficacy, objective sleep, and the severity of OSA. In the future, we can combine with the Health Belief Models to formulate an individualized intervention plan based on patients’ self-efficacy.

## 1 Introduction

Obstructive sleep apnea (OSA) is a common chronic condition marked by the partial or complete collapse of the upper airway during sleep. This disorder leads to various pathophysiological effects (1), such as fragmented sleep, intermittent low oxygen levels, excessive daytime sleepiness, and altered sleep patterns. Strong epidemiological studies have established OSA as an independent risk factor for both the occurrence and recurrence of strokes (2–4). Additionally, individuals with OSA often experience poorer functional recovery, longer hospital stays, and a higher risk of mortality (5). The interaction of undiagnosed and untreated obstructive sleep apnea (OSA) presents a significant public health challenge, as it places an undue burden on healthcare systems and socioeconomic structures. Positive airway pressure (PAP) therapy is the primary treatment for OSA, working by stabilizing the upper airway through the delivery of pressure via a mask during sleep. Research from randomized controlled trials has shown that consistent use of PAP therapy for at least 4 h each night can improve cognitive performance, enhance sleep quality, reduce excessive daytime sleepiness, and support neurological recovery (6–9), However, the effectiveness of this treatment heavily relies on patient adherence, which is notably lower among stroke survivors compared to those with OSA who have not experienced a stroke (10).

The Health Belief Model (HBM), a value-expectancy theory grounded in social cognitive principles (11), elucidates health behavior determinants through dual appraisal processes: threat perception involving disease susceptibility and severity evaluations, and behavioral assessment weighing intervention benefits against implementation barriers (12). With the advancement of HBM, this study provides a compelling illustration of the significant role that health education plays in enhancing the effectiveness and benefits of patients’ perceived health behaviors. For researchers, it offers a valuable theoretical framework. Moreover, it serves as a foundation for evaluating the factors influencing patients’ non-adherence behavior and devising personalized intervention plans (13). While existing research has established a dose-dependent relationship between Health Belief Model (HBM) constructs and Positive Airway Pressure (PAP) adherence in general populations with Obstructive Sleep Apnea (OSA), this conceptual framework has been notably underexplored in populations with post-stroke OSA.

To address the significant gap in evidence, this study utilizes the Health Belief Model (HBM) to thoroughly assess patient perceptions across five important areas: perceived susceptibility, perceived severity, beliefs about treatment effectiveness, thresholds for perceived benefits, and hierarchies of perceived barriers. These findings aim to create a solid empirical framework that can guide the development of tailored behavioral interventions, specifically designed to improve adherence to respiratory therapy among patients at high risk.

## 2 Materials and methods

### 2.1 Study population

This is a cross-sectional study using convenience sampling method to facilitate the sampling of patients who were outpatients and inpatients in the Department of Neurology of a tertiary-level hospital in Yangzhou City from November 15, 2023, to May 15, 2024, and who met the inclusion and exclusion criteria.

Inclusion criteria: (1) Stroke patients were diagnosed according to the diagnostic criteria revised by the Fourth National Academic Conference on Cerebrovascular Disease of the Chinese Medical Association in 1995; (2) Age≥18 years, clear consciousness, cooperation in examination, and absence of speech and hearing disorders; (3) First diagnosed by polysomnography and AHI > 5 beats/h; (4) The patients or their families gave informed consent.

Exclusion criteria: (1) Presence of serious complications with impaired consciousness; (2) Presence of psychiatric disease or previous history of the psychiatric disease; (3). Taking sedative drugs; (4) Combined asthma, chronic obstructive pulmonary disease; 5. Combined neuromuscular disease-induced sleep apnea; 6. Incomplete or lost data.

### 2.2 Research tools

1. General information questionnaire including the patient’s age, sex, education level, marital status, area of residence, work status, nature of occupation, financial situation, whether he/she has medical insurance, whether he/she has underlying diseases, and bad hobbies.
2. The Pittsburgh Sleep Quality Index (PSQI) (14): Crombach’s *α* was0.88, used to evaluate the quality of sleep in the last 1 month, including sleep quality, time to fall asleep, total sleep time, sleep efficiency, sleep disorders, hypnotic drug application, daytime dysfunction of a total of 7 dimensions, each item scored 0–3 points, the total score of 21 points, the higher the score, the worse the subjective sleep quality.
3. Epworth Sleepiness Scale (ESS) (15): can be used to evaluate the degree of subjective drowsiness in adults, including reading in a sitting position, watching TV, sitting still in public places, traveling by car for 1 h without resting, resting in bed in the afternoon if conditions permit, sitting still and talking with others, sitting still after meals without drinking alcohol at lunch, and stopping the car for a few minutes in traffic jams for a total of 8 items, with a score of 0–3 for each item and a total score of 24, with the higher the score, the more serious the tendency to drowsiness, and the score of >10 being the presence of excessive daytime sleepiness. >10 was classified as the presence of excessive daytime sleepiness, and the Crombach’s *α* of the scale was 0.88.
4. Health beliefs: In 2003, Weaver et al. developed the Self-efficacy Measure for Sleep Apnea (SEMSA) (16), which assesses the social perception of OSA patients towards the disease and PAP treatment. The instrument employs three dimensions: risk perception, outcome expectation and self-efficacy. Each dimension is evaluated using a 4-point Likert scale. Higher scores in each dimension indicate greater knowledge of OSA, higher expectations for PAP treatment, higher self-efficacy and stronger health beliefs. The Crombach’s *α* was 0.89, while the Crombach’s α coefficient of each dimension ranged from 0.75 ~ 0.90.
5. Hospital Anxiety and Depression Scale (HADS) (17): Evaluates the state of psychological functioning in the last month, and is mainly applied to screen for anxiety and depression among patients in general hospitals. The HADS consists of a total of 14 entries, of which 7 entries rate depression and 7 entries rate anxiety, with 0–7 being normal; 8–10 being mild depression/anxiety; 11–15 being moderate depression/anxiety; and ≥16 being severe depression/anxiety.
6. Polysomnography: All participants underwent overnight polysomnography (PSG) under video using a SOMNOscreen polysomnography monitor, including 6-channel EEG, 2-channel ocular electro-oculogram tracing method, and chin muscle electromyography. In addition, recordings included electrocardiogram, respiratory flow (nasal and oral thermistors), nasal force-pressure tubes, respiratory effort based on thoracic and abdominal respiratory inductance volume tracings, oximetry (transmitter type refers to photovoltaic volumetric pulse wave), snoring microphone, body position (gravity-based electrical transducer), and tibialis anterior muscle electromyography. All sleep reports were interpreted by professional technicians and physicians with a Master’s degree or higher, with an international Registered Polysomnographic Technician Qualification (RPSGT), and engaged in the study of sleep-related disorders, and all data were processed and analyzed according to the AASM interpretation manual (18). Sleep staging was assessed with reference to the American Academy of Sleep Medicine (AASM) guidelines for sleep and related events (19), which can be classified into 4 stages: Wakefulness (W), non-rapid eye movement sleep 1 (NREM1), non-rapid eye movement sleep 2 (NREM2), non-rapid eye movement sleep 3 (NREM3), and rapid eye movement sleep (REM). Total sleep time, wake after sleep onset (WASO), sleep latency (SL), sleep efficiency (SE), and percentage of each sleep stage. Apnea Hypoventilation Index (AHI) is the average number of apneas and hypoventilations per hour during sleep. Lowest oxygen saturation in blood (LSpO2) was defined as the lowest value of percentage of blood oxygen during sleep. Oxygen descent index (ODI) was defined as the number of hourly decreases in blood oxygen ≥3% from baseline during sleep (20).
7. Definition of Adherence: Ventilator usage data were collected by extracting records from the built-in data storage module of the home ventilator. Treatment compliance was defined as the utilization of the device for at least 4 h per night on ≥70% of monitored nights (21), Patients were considered to be adherent if they used respiratory therapy for 4 h or more per night on at least 63 of the 3 months (90 days) monitored.

### 2.3 Statistical methods

SPSS 26.0 was used for data analysis, and the measurement information was expressed as ( $\bar{x}\pm s$ ); Independent samples t-test for comparison between two groups; Pearson correlation analysis was used for correlation analysis between variables; and logistic regression analysis was used for the influencing factors of PAP adherence, *p* < 0.05 was considered statistically significant.

## 3 Results

### 3.1 Study population characteristics and adherence profile

**Figure 1:**
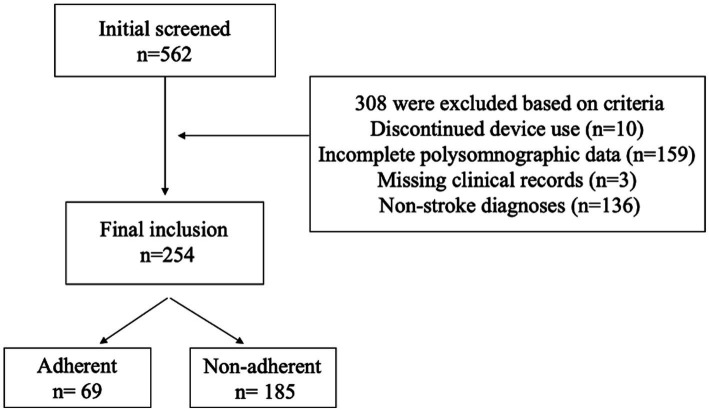
Participant flowchart.

The study initially screened 562 participants who underwent diagnostic polysomnography, 308 individuals were excluded based on predefined criteria: 10 discontinued device use, 159 exhibited incomplete polysomnographic data, 3 lacked essential clinical documentation, and 136 received non-stroke diagnoses. Consequently, the final analytical cohort comprised 254 stroke patients with confirmed obstructive sleep apnea (OSA), as detailed in Figure 1. These patients were stratified based on their adherence to positive airway pressure (PAP) therapy, defined by standard criteria, As shown in Table 1, there were notable demographic differences between the adherent group (*n* = 69, 27.2%) and the non-adherent group (*n* = 185, 72.8%). Specifically, adherent patients were younger, with an average age of 62.6 years compared to 67.2 years for non-adherent patients, and this difference was statistically significant (*p* = 0.00). Additionally, adherent patients had a lower body mass index (BMI). Furthermore, adherence rates were higher among urban residents, with 62% adherence compared to 38% in rural residents, which was also statistically significant (*p* = 0.04).

**Table 1:** Comparison of general information of patients with stroke with OSA.

Categories	Compliance (*n* = 69)	Non-compliance (*n* = 185)	t	*P*
Age	62.6 ± 11.3	67.2 ± 10.6	−2.997	<0.05
Gender			2.115	0.15
Man	50	116		
Women	19	69		
BMI (kg/m^2^)			192.161	<0.05
≤18.5	3	0		
18.6 ~ 22.9	50	0		
23.0 ~ 24.9	16	52		
≥25.0	0	133		
Educational level			7.965	0.47
Junior high school and below	49	153		
Senior high school	11	25		
College	3	2		
Undergraduate and above	6	5		
Residence			4.886	0.18
Live alone	2	13		
Spouse	58	161		
Children	4	4		
Spouse and children	51	7		
Current address			4.379	0.04
Countryside	26	97		
Urban	43	88		
Working conditions			9.874	0.01
Incumbency	13	12		
Retirement	56	169		
Out of work	0	4		
Nature of work			3.106	0.08
Brainwork	21	37		
Physical labor	48	148		
Medical insurance			1.132	0.29
None	0	3		
With	69	182		
monthly salary			3.151	0.21
<5,000	59	170		
5,000–10,000	10	14		
>10,000	0	1		
Medications			10.072	0.02
None	9	6		
1 type	5	18		
2–3 types	53	159		
3 types	2	2		
Hypertensive			0.643	0.42
None	19	42		
With	50	143		
Diabetes				
None	45	125	0.125	0.72
With	24	60		
Cardiology			2.174	0.14
None	64	159		
With	5	26		
Smoking			1.971	0.16
None	47	147		
With	22	43		
Drinking			3.694	0.06
None	50	154		
With	19	31		

### 3.2 Univariate predictors of PAP adherence

In this study, adherence to PAP therapy in stroke patients with OSA showed no significant association with subjective sleep quality, daytime sleepiness, or anxiety and depression status (*p* > 0.05). However, significant correlations were observed with several objective measures, including the sleep apnea self-efficacy scale (perceived risk, expected treatment effect, self-assessment, and total score), Modified Rankin Scale (MRS) scores at 3 months, and polysomnographic parameters such as total sleep time, sleep efficiency, N1/N2/N3 sleep percentages and durations, apnea-hypopnea index (AHI), longest hypopnea duration, minimum oxygen saturation, and oxygen desaturation index (ODI) ≥ 3 (*p* < 0.05), as detailed in Table 2. One-way analysis of variance further revealed that the adherence group exhibited higher scores across all dimensions of the self-efficacy scale, improved sleep quality, and greater OSA severity compared to the non-adherence group. Additionally, the adherence group demonstrated a higher frequency of oxygen desaturation events (ODI ≥ 3), underscoring the role of objective sleep metrics in predicting treatment compliance.

**Table 2:** Factors influencing PAP adherence in patients with stroke with OSA.

Variables	Compliance (*n* = 69)	Non-compliance (*n* = 185)	t	*P*
ESS	8.58 ± 6.58	7.44 ± 6.04	−1.302	0.17
PSQI	8.88 ± 4.42	8.74 ± 4.75	−0.226	0.82
Sleep quality	1.49 ± 0.90	1.38 ± 0.89	−0.908	0.37
Bedtime	1.38 ± 1.26	1.35 ± 1.28	−0.142	0.89
Sleep duration	1.26 ± 1.07	1.36 ± 1.15	0.637	0.53
Sleep efficiency	1.38 ± 1.34	1.49 ± 1.25	0.639	0.52
Sleep disorder	1.39 ± 0.52	1.35 ± 0.53	−0.535	0.59
Soporific drug	0.38 ± 0.91	0.34 ± 0.91	−0.324	0.75
Daytime dysfunction	1.62 ± 1.31	1.47 ± 1.29	−0.837	0.40
HADS-A	1.87 ± 1.20	2.14 ± 2.52	0.788	0.43
HADS-D	4.49 ± 0.72	4.16 ± 2.62	−1.050	0.30
Risk perception	17.60 ± 3.9	13.9 ± 0.80	12.289	<0.05
Expected results of treatment	22.0 ± 5.9	17.9 ± 0.70	9.308	<0.05
Self-assessment	19.7 ± 6.6	18.0 ± 1.30	3.250	<0.05
Total health belief score	59.3 ± 12.5	49.90 ± 1.80	9.997	<0.05
MRS	0.54 ± 0.87	0.58 ± 0.85	0.394	0.69
MRS (3 months)	0.25 ± 0.47	0.56 ± 0.73	−3.232	0.00
Objective sleep conditions				
Total sleep time (minutes)	407.40 ± 128.80	358.80 ± 131.00	2.642	0.01
Sleep efficiency (%)	76.20 ± 15.40	70.70 ± 17.60	2.284	0.02
N1 sleep percentage	11.60 ± 10.90	9.20 ± 9.40	1.704	0.09
N2 sleep percentage	62.10 ± 14.70	14.70 ± 14.80	2.668	0.01
N3 sleep percentage	8.00 ± 8.10	14.70 ± 14.80	−3.567	<0.05
N1 duration	44.50 ± 44.60	30.00 ± 28.60	3.048	0.00
N2 duration	255.00 ± 98.10	200.90 ± 93.80	4.037	<0.05
N3 duration	32.80 ± 35.80	52.40 ± 56.30	−2.687	0.01
AHI	33.30 ± 20.10	24.30 ± 19.60	3.238	0.00
Maximum hypoventilation (s)	99.00 ± 20.30	56.30 ± 35.20	2.551	0.01
Minimum oxygen saturation (%)	82.40 ± 8.30	85.00 ± 9.10	−2.059	0.04
ODI	37.20 ± 19.30	24.90 ± 21.10	2.929	0.00

PSQI, The Pittsburgh Sleep Quality Index; ESS = Epworth Sleepiness Scale; HADS-A, Hospital Anxiety and Depression Scale Anxiety; HADS-D=Hospital Anxiety and Depression Scale Depression; MRS, Modified Rankin Scale; AHI, apnea hypopnea index; ODI, Decrease in oxygen saturation≥3 index.

### 3.3 Pearson correlation analysis

Bivariate Pearson correlation analyses revealed significant associations between treatment adherence and multiple clinical parameters in our cohort (Table 3). The data demonstrated inverse correlations of adherence with age (r = −0.186, *p* = 0.03) and body mass index (BMI; r = −0.828, *p* < 0.05), indicating reduced compliance in older patients and those with higher adiposity. Conversely, positive correlations emerged between adherence and health belief constructs Risk perception (r = 0.579, *p* < 0.00), Treatment benefit expectations (r = 0.369, *p* < 0.05), Self-efficacy (r = 0.247, *p* < 0.05), Total health belief score (r = 0.512, *p* < 0.05) and Sleep architecture metrics Total sleep time (r = 0.164, *p* = 0.01), N1 duration (r = 0.189, *p* = 0.00), N2 duration (r = 0.246, *p* < 0.05). These findings suggest adherence behaviors are simultaneously influenced by psychosocial cognition (health beliefs) and physiological sleep patterns while being constrained by demographic factors (age/BMI).

**Table 3:** Analysis of PAP adherence correlation in patients with stroke combined with OSA.

	Compliance	Age	BMI	Risk perceived	Expected effect of treatment	self-assessment	Total health belief score	Total sleep time (min)	N1 sleep percentage	N1 duration	N2 duration
Compliance	1										
Age	−0.186^**^	1									
BMI	−0.828^**^	0.155^*^	1								
Risk perception	0.579^**^	−0.210^**^	−0.601^**^	1							
Expected effect of treatment	0.369^**^	−0.219^**^	−0.472^**^	0.576^**^	1						
self-assessment	0.247^**^	−0.083	−0.12	0.078	0.251^**^	1					
Total health belief score	0.512^**^	−0.226^**^	−0.507^**^	0.674^**^	0.836^**^	0.670^**^	1				
Total sleep time (min)	0.164^**^	0.014	−0.054	0.095	0.067	0.168^**^	0.153^*^	1			
N1 sleep percentage	0.106	0.04	−0.1	0.056	0.073	0.132^*^	0.124^*^	−0.201^**^	1		
N1 duration	0.189^**^	0.033	−0.157^*^	0.117	0.115	0.191^**^	0.197^**^	0.271^**^	0.785^**^	1	
N2 duration	0.246^**^	−0.061	−0.092	0.121	0.065	0.189^**^	0.172^**^	0.784^**^	−0.289^**^	0.089	1

*p < 0.05, **p < 0.001.

### 3.4 Logistic regression analysis

The multivariable logistic regression model integrating significant univariate and correlation predictors revealed distinct determinants of PAP adherence (Table 4). Health belief constructs exhibited robust dose–response relationships, with perceived risk (OR = 2.22, 95% CI = 1.74–2.83; *p* < 0.05), treatment benefit expectations (OR = 1.32, 95% CI = 1.23–1.41; *p* < 0.05), self-management capacity (OR = 1.17, 95% CI = 1.06–1.29; *p* = 0.00), and the composite health belief score (OR = 1.19, 95% CI = 1.13–1.26; *p* < 0.05) all significantly associated with adherence. Polysomnographic measures also contributed, including total sleep time (OR = 1.00, 95% CI = 1.00–1.01; *p* = 0.01), N1 sleep proportion (OR = 1.01, 95% CI = 1.00–1.02; *p* = 0.03), apnea-hypopnea index (AHI) (OR = 1.04, 95% CI = 1.02–1.06; *p* = 0.00), and oxygen desaturation index (ODI) (OR = 1.03, 95% CI = 1.01–1.05; *p* = 0.01). Furthermore, post-PAP sleep efficiency improvement (OR = 1.00, 95% CI = 1.00–1.04; *p* = 0.02) emerged as a significant treatment-response marker, highlighting the multifaceted predictors of adherence.

**Table 4:** Logistic regression analysis of PAP adherence in patients with stroke with OSA.

Variables	β	SE	Wald $\;x^{2}$	OR	95 per cent CI	*P*
Risk perception	0.80	0.12	41.50	2.22	1.74 ~ 2.83	<0.05
Expected results of treatment	0.21	0.04	30.24	1.23	1.23 ~ 1.41	<0.05
Self-assessment	0.16	0.05	9.35	1.17	1.06 ~ 1.29	0.00
Total health belief score	0.18	0.03	38.77	1.19	1.13 ~ 1.26	<0.05
Objective sleep conditions						
Total sleep time	0.00	0.00	6.66	1.00	1.00 ~ 1.01	0.01
Sleep efficiency	0.02	0.01	5.34	1.00	1.00 ~ 1.04	0.02
N1 Duration	0.01	0.00	4.60	1.01	1.00 ~ 1.02	0.03
N2 Duration	0.01	0.00	13.05	1.01	1.00 ~ 1.01	<0.05
AHI	0.039	0.01	10.29	1.04	1.02 ~ 1.06	0.05
Maximum duration of hypoventilation (s)	0.02	0.01	6.15	1.02	1.00 ~ 1.03	0.01
ODI	0.03	0.01	7.49	1.03	1.01 ~ 1.05	0.01

AHI, apnea hypopnea index; ODI, Decrease in oxygen saturation≥3 index.

## 4 Discussion

While Positive Airway Pressure (PAP) therapy demonstrates established efficacy in stroke management, optimizing treatment adherence persists as a critical clinical challenge. This study investigation, based on the Health Belief Model (HBM), elucidates modifiable psychological and behavioral determinants underlying PAP adherence patterns in stroke patients, providing a theoretical foundation for personalized intervention development.

This study conducted a multidimensional analysis of compliance with positive airway pressure (PAP) therapy among patients with stroke combined with obstructive sleep apnea (OSA). Among the 254 included patients, only 27.2% (69 cases) met the compliance criteria (usage time >4 h/night and coverage rate ≥70%), a rate significantly lower than the 40–50% baseline reported in the general OSA population (22–24). This discrepancy may be attributed to the unique demographic profile of our cohort: middle-aged and elderly stroke patients with limited health literacy. Their complex cultural background, low educational attainment (60.23% with primary school education or below), reduced economic status (66.9% with monthly household income <5,000 RMB), and high rural residency (38.2%) likely contributed to insufficient health literacy to address practical challenges in PAP use. These patients may also lack full understanding of OSA risks and PAP benefits, while deficient disease awareness might further compromise adherence (25, 26). Neurological deficits and cognitive impairment in elderly stroke patients could exacerbate treatment adherence difficulties, as traditional educational interventions may fail due to impaired information processing capacity (27).

Excessive daytime sleepiness (EDS), a hallmark OSA symptom and predictor of severe apnea (28), showed no significant association with PAP compliance in our analysis. Baseline EDS severity (Epworth Sleepiness Scale: 8.58 ± 6.58 vs. 7.44 ± 6.04 for compliant vs. non-compliant groups, *p* > 0.05) and subjective sleep quality metrics were unrelated to adherence outcomes, contrasting with prior reports linking symptom relief to sustained PAP use (22). However, consistent with existing evidence (29, 30), compliant patients demonstrated significant ESS score reductions post-PAP, suggesting longitudinal ESS changes may predict long-term adherence. The apnea-hypopnea index (AHI), as the gold standard for OSA severity, strongly correlated with compliance (31). Patients with AHI > 30 events/h exhibited higher treatment motivation, likely due to more pronounced symptoms. Delijaj et al. (32) identified nocturnal arousals and sleep fragmentation via remote monitoring as independent predictors of reduced PAP adherence. Our findings align with this mechanism: compliant patients showed improved sleep quality, extended total sleep time, enhanced sleep efficiency, stabilized light sleep, and reduced periodic limb movements.

Health beliefs represent modifiable targets for improving PAP adherence in stroke-OSA patients (33). Our results demonstrate that respiratory therapy compliance is shaped by disease perception, outcome expectations, and self-efficacy cognitive domains critical to treatment initiation. Discrepancies between anticipated and actual treatment experiences may drive non-adherence (33, 34), potentially exacerbating neurological deficits, sleep architecture deterioration, and stroke recurrence risk (35). Healthcare professionals, particularly nurses, should deliver tailored education to enhance patients’ understanding of health beliefs related to PAP. Behavioral strategies promoting positive treatment attitudes should be prioritized. Proposed interventions aiming to assess and educate patients about health beliefs should encompass: (1) OSA pathophysiology and health impacts, (2) available OSA therapies with emphasis on PAP mechanisms/advantages, (3) side effect management, and (4) evidence-based adherence strategies. This educational framework must be integrated throughout the OSA care continuum from initial diagnosis and sleep laboratory assessment to long-term treatment to reinforce PAP understanding and proper use, ultimately achieving sustained adherence improvement.

Study limitations warrant cautious interpretation. First, the single-center cross-sectional design and predominant inclusion of elderly participants (mean age 62.6 ± 11.3 and 67.2 ± 10.6 years) may limit generalizability to younger stroke populations - a demographic experiencing rising OSA incidence. Second, our 3-month observation window precludes assessment of long-term adherence determinants. Future investigations should incorporate multicenter longitudinal designs with extended follow-up periods (≥12 months) to capture dynamic compliance patterns. Additionally, the study’s use of convenience sampling may limit generalizability, as participants may not be fully representative of the broader population of stroke-associated OSA patients. Future studies should prioritize serial enrollment to mitigate.

## 5 Conclusion

Health beliefs are modifiable factors that significantly influence PAP adherence in stroke patients with OSA. Integrating the HBM with patients’ self-efficacy offers a promising approach to developing personalized adherence-enhancing strategies, ultimately improving clinical outcomes in this high-risk population.

## Data Availability

The raw data supporting the conclusions of this article will be made available by the authors, without undue reservation.
